# *Editorial Commentary*: Out of Africa: Links Between Invasive Nontyphoidal *Salmonella* Disease, Typhoid Fever, and Malaria

**DOI:** 10.1093/cid/cit803

**Published:** 2013-12-13

**Authors:** Calman A. MacLennan

**Affiliations:** Novartis Vaccines Institute for Global Health, Siena, Italy; Medical Research Council Centre for Immune Regulation, School of Immunity and Infection, College of Medicine and Dental Sciences, University of Birmingham, United Kingdom

**Keywords:** *Salmonella*, typhoid, malaria, Africa, Tanzania

**(See the Major Article by Biggs et al on pages 638–47.)**

The report by Biggs et al in the current issue of *Clinical Infectious Diseases* provides important insight into relationships between the 2 main forms of invasive *Salmonella* disease and the major childhood infectious disease burden in sub-Saharan Africa—malaria. It is the latest in a series of carefully conducted clinical studies from Tanzania from the research groups of John Crump and Hugh Reyburn. The study combines blood culture and clinical data from 2 major hospitals located some 250 km apart in very different settings: the Kilimanjaro Christian Medical Centre (KCMC), at 890 m at the foot of Mount Kilimanjaro in Moshi, where malaria transmission is low and seasonal, and Teule Hospital, at 96 m near the coast in Muheza, where malaria transmission is intensive and perennial.

Invasive *Salmonella* disease can be divided broadly into enteric fever, principally caused by *Salmonella enterica* serovars Typhi and Paratyphi A, and invasive nontyphoidal *Salmonella* (iNTS) disease, mainly caused by *Salmonella* Typhimurium and Enteritidis. Enteric fever is a particular problem in Southeast Asia [1], where iNTS disease is relatively uncommon. By contrast, iNTS disease is responsible for a much larger disease burden than enteric fever in sub-Saharan Africa, causing >100 000 deaths a year. In many African countries, nontyphoidal *Salmonella* (NTS) is the commonest cause of bacteremia [2], although in recent years, there has been a growing number of reports of invasive disease caused by *S*. Typhi in the region. It is currently uncertain what is driving the evolving epidemiology of invasive *Salmonella* disease in sub-Saharan Africa. The sites in Tanzania where this study was conducted represent 2 locations in Africa where both iNTS disease and typhoid can be studied.

The authors investigated bacteremia among febrile children admitted to both hospitals. There was no difference in the isolation rates of *Streptococcus pneumoniae* and *Escherichia coli*, 2 commonly isolated bacterial pathogens, between the sites. However, bacteremia at Teule Hospital was twice as common as at KCMC and this difference is due to the higher incidence of iNTS disease at Teule. Half of all pathogenic isolates from that site were NTS, whereas only 1 case of NTS bacteremia was detected at KCMC. In contrast, *S*. Typhi accounted for 3% of culture isolates at Teule and a third at KCMC.

These findings suggest the presence of a factor that exerts a strong influence on the 2 types of invasive *Salmonella* disease. Malaria is the obvious candidate. Malaria parasites were present in more than half of febrile children admitted at Teule, but only 2% at KCMC. Three-quarters of iNTS disease cases at Teule were associated with malaria or recent malaria. By multivariate analysis, iNTS disease was significantly associated with younger age, recent malaria, acute severe malnutrition, and severe anemia.

A constant challenge in the interpretation of observational studies is determining which associations are causal. A number of well-recognized risk factors are associated with iNTS disease in African children, including malaria. The association between malaria and *Salmonella* disease in Africa was first reported by Giglioli in British Guiana in the 1920s, albeit with *S*. Paratyphi C [3], and then again by Mabey et al in the Gambia in 1987 [4]. Subsequently, the clinical link between the 2 diseases has become well established. There have been recent reports of falling levels of overall bacteremia and iNTS disease in locations in sub-Saharan Africa, where malaria levels are declining [5]. Nevertheless, persistently high levels of iNTS disease are still found across much of the continent [2]. Other well-recognized clinical associations with iNTS disease are young age, malnutrition, human immunodeficiency virus (HIV), and anemia, often presenting as severe malarial anemia [2].

A relative importance for malaria in the etiology of iNTS disease in the Tanzanian setting is suggested by the finding that severe acute malnutrition and HIV disease are more common among febrile children at KCMC than at Teule. The Crump/Reyburn team previously reported a close link between iNTS disease and HIV infection among adult inpatients at KCMC, as well as a significant negative association between HIV infection and enteric fever [6]. The lack of association between HIV infection and iNTS bacteremia in the current study could be due to the low prevalence of HIV infection among children at Teule.

The mechanisms underlying the association between iNTS disease and malaria have been explored in murine coinfection models. They include a reduction in neutrophil oxidative burst activity, attributed to the induction of heme oxygenase-1 secondary to hemolysis [7], reduced levels of interleukin 12 (IL-12) [8], and increased levels of interleukin 10 (IL-10) [9]. The latter 2 possibilities are consistent with the apparently paradoxical relative lack of iNTS disease in children with high parasite counts in the current study. High malaria parasite counts are common in cerebral malaria where there is marked upregulation of the inflammatory/T helper 1 group of cytokines, including interferon-γ, tumor necrosis factor–α, and IL-12, which are moderated by the production of IL-10.

Typhoid fever in this study was associated with older age and negatively associated with current malaria. No patient with typhoid fever was infected with HIV or had had recent malaria. However, whereas there were 163 episodes of iNTS disease in the study, there were only 17 of typhoid fever, partly because there were almost 10-fold fewer children from KCMC than from Teule. The small number of typhoid fever cases makes it difficult to draw firm conclusions from this study about its emergence in Africa.

From this and other studies, there appears to be some mutual exclusivity between iNTS disease and typhoid fever in Africa. Typhoid fever is usually uncommon in settings where iNTS disease and malaria are prevalent. A study from 2 sites in Kenya similarly found high levels of iNTS disease and low levels of typhoid fever in a high malaria transmission rural setting, and the converse in a low malaria transmission urban setting [10]. As with the current study, differential malaria endemicity was proposed as the underlying reason for this epidemiology. Recent data from Malawi indicate a sustained decrease in iNTS disease since 2003 with a rise in typhoid fever from 2011, such that in 2012 *S*. Typhi was more commonly isolated from blood than iNTS [11]. The changes occurred without a clear change in malaria transmission, although half a million Malawians have started antiretroviral therapy since 2003. This is evidence that other factors, particularly HIV infection, are relevant to the occurrence and etiology of invasive *Salmonella* disease in Africa.

What additional factors could have a bearing on the occurrence of invasive *Salmonella* disease in Africa? Although the clinical data have been well analyzed in this study, there is a lack of information about host immunity to *Salmonella* in this setting, the actual bacteria isolates responsible for these infections, and possible environmental factors that could affect *Salmonella* transmission. The acquisition of antibodies against NTS with age is associated with a fall in incidence of iNTS disease among Malawian children [12], and differences in acquired antibody and T-cell immunity to both forms of *Salmonella* at the 2 hospitals could have an important impact on the amount of disease seen. There is general lack of information about temporospatial differences in patient immunity against different forms of *Salmonella* across Africa. Such knowledge could prove key to making sense of the emerging epidemiology of invasive *Salmonella* disease and help predict *Salmonella* epidemics.

On the pathogen side of the host–pathogen interface, details about the antibiotic resistance of the different *Salmonella* isolates and the serovar identities of the iNTS isolates (Typhimurium, Enteritidis, or other) are not available in this report. The former could be particularly significant given the high proportion of children who had received antibiotics prior to admission. The NTS epidemic in sub-Saharan Africa has been associated with the emergence of a new pathovar of *S*. Typhimurium characterized by the multilocus sequence type ST313 [13]. Similarly, the emergence of *S*. Typhi in sub-Saharan Africa has been associated with the spread of the H58 haplotype from Southeast Asia [14]. It is important to determine whether these strains are circulating in Tanzania. Finally, problems with clean water security and basic sanitation can underpin the spread of *S*. Typhi [1]. Some of the earliest reports of the emergence of typhoid fever in the region were from the Kibera slum of Nairobi [10].

What are the implications for the management of invasive *Salmonella* disease? Clearly, a high index of suspicion for iNTS disease is required among febrile children who are “slide negative” for malaria parasites in Africa, particularly if they have recently had an episode of malaria. Vigilance needs to be maintained for the emergence of typhoid fever in Africa, especially in sites where the prevalence of malaria and/or iNTS disease is falling. Finally, it cannot be assumed that all invasive *Salmonella* disease in Africa is due to NTS or *S*. Typhi. Therefore, clinical interventions, including new vaccines, need to be developed against both types of *Salmonella* disease.

In summary, the study by Biggs et al provides useful insights into the interrelatedness of iNTS disease, typhoid fever, and malaria in Africa. The main conclusion is the confirmation of the close association between iNTS disease and malaria among African children to a level where a role in the etiology of iNTS disease is supported. Secondary conclusions are the lack of such an association with typhoid fever, and an apparent partial mutual exclusivity between iNTS disease and enteric fever in Africa. Combined with the Tanzanian groups' previously reported work in relation to HIV infection and invasive *Salmonella* disease, the 2 studies reinforce the premise that iNTS disease is a problem among African children where malaria transmission is high and among adults where HIV infection is prevalent. In areas where these comorbidities are uncommon or incidence levels are falling, typhoid fever threatens to emerge, particularly where clean water security is low and sanitation is poor.

To further understand the relationship between these 3 diseases, longitudinal studies at multiple sites are needed to track their evolving epidemiology over time. To bring mechanistic insight to this epidemiology, such clinical work needs to be supported by laboratory work to appraise host immunity carefully, both among affected individuals and background communities, as well as to determine the genomic, molecular, and clinical microbiology of the disease-causing isolates. Such work will require an increased investment in Africa. This is essential to improve clinical practice and guide the development of new medical interventions against a group of diseases that continue to be responsible for a heavy burden of morbidity and mortality.

## References

[1] Crump JA, Luby SP, Mintz ED (2004). The global burden of typhoid fever. Bull World Health Organ.

[2] MacLennan CA, Levine MM (2013). Invasive nontyphoidal *Salmonella* disease in Africa: current status. Expert Rev Anti Infect Ther.

[3] Giglioli G (1929). Paratyphoid C, an endemic disease in British Guiana. J Hyg.

[4] Mabey DC, Brown A, Greenwood BM (1987). *Plasmodium falciparum* malaria and *Salmonella* infections in Gambian children. J Infect Dis.

[5] Scott JA, Berkley JA, Mwangi I (2011). Relation between falciparum malaria and bacteraemia in Kenyan children: a population-based, case-control study and a longitudinal study. Lancet.

[6] Crump JA, Ramadhani HO, Morrissey AB (2011). Invasive bacterial and fungal infections among hospitalized HIV-infected and HIV-uninfected adults and adolescents in northern Tanzania. Clin Infect Dis.

[7] Cunnington AJ, de Souza JB, Walther M, Riley EM (2012). Malaria impairs resistance to *Salmonella* through heme- and heme oxygenase-dependent dysfunctional granulocyte mobilization. Nat Med.

[8] Roux CM, Butler BP, Chau JY (2010). Both hemolytic anemia and malaria parasite-specific factors increase susceptibility to nontyphoidal *Salmonella enterica* serovar Typhimurium infection in mice. Infect Immun.

[9] Lokken KL, Mooney JP, Butler BP (1995). Malaria parasite-induced IL-10 contributes to non-typhoid *Salmonella* bacteremia [abstract S1:7].

[10] Tabu C, Breiman RF, Ochieng B (2012). Differing burden and epidemiology of non-Typhi *Salmonell*a bacteremia in rural and urban Kenya, 2006-2009. PLoS One.

[11] Feasey NA, Heyderman RS, Gordon MA (2013). *Salmonella* blood-stream infection in Malawi: a fall in nontyphoidal *Salmonella* and an outbreak of typhoid [abstract 88].

[12] MacLennan CA, Gondwe EN, Msefula CL (2008). The neglected role of antibody in protection against bacteremia caused by nontyphoidal strains of *Salmonella* in African children. J Clin Invest.

[13] Okoro CK, Kingsley RA, Connor TR (2012). Intracontinental spread of human invasive *Salmonella* Typhimurium pathovariants in sub-Saharan Africa. Nat Genet.

[14] Kariuki S, Revathi G, Kiiru J (2010). Typhoid in Kenya is associated with a dominant multidrug-resistant *Salmonella enterica* serovar Typhi haplotype that is also widespread in Southeast Asia. J Clin Microbiol.

